# Synthetic peptide V11A reduces bacterial load and inflammation in pneumococcal meningitis

**DOI:** 10.3389/fcimb.2025.1686525

**Published:** 2026-02-09

**Authors:** Janine Lux, Maria Erhardt, Linus Rechsteiner, Hannah Agnew, Robert Hoepner, Roger Koller, Denis Grandgirard, Stephen L. Leib, Lucy J. Hathaway

**Affiliations:** 1 Institute for Infectious Diseases, Faculty of Medicine, University of Bern, Bern, Switzerland; 2 Graduate School for Cellular and Biomedical Sciences, University of Bern, Bern, Switzerland; 3 Department of Neurology, Bern University Hospital and University of Bern, Bern, Switzerland; 4 Multidisciplinary Center for Infectious Diseases, University of Bern, Bern, Switzerland

**Keywords:** cerebrospinal fluid, *Klebsiella pneumoniae*, meningitis, peptide, V11A, Streptococcus pneumoniae

## Abstract

**Introduction:**

Pneumococcal meningitis, caused by Streptococcus pneumoniae, is exacerbated by factors released during bacterial lysis, triggering inflammation and damage to host cells. Bacteriolytic antibiotics increase inflammation meaning that there is a high unmet medical need for non-bacteriolytic antimicrobials as alternative therapeutic options. Previously, an 11-amino acid peptide was discovered in the secretome of Klebsiella pneumoniae, named V11A, and was found to inhibit growth of S. pneumoniae in a bacteriostatic and species-specific manner in vitro.

**Methods and Results:**

Here it is shown that peptide V11A reduced the growth of not only S. pneumoniae spiked into human cerebrospinal fluid (hCSF) of non-meningitis donors but also S. pneumoniae present naturally in hCSF of a patient with pneumococcal meningitis (hmCSF). In an infant rat model of pneumococcal meningitis, V11A not only reduced the number of S. pneumoniae bacteria in CSF and blood but also reduced the concentration of cytokines GRO/KC/CINC-1 (an interleukin-8 (IL-8)-like cytokine in rats) and IL-10 in CSF.

**Discussion:**

Our results support the potential of therapeutic peptide to reduce the bacterial burden and mitigate the inflammatory response in pneumococcal meningitis.

## 1 Introduction

Pneumococcus is a major human pathogen that causes a global health burden due to invasive pneumococcal diseases including meningitis (Müller et al., 2022; Ozisik, 2025). *Streptococcus pneumoniae* causes over 50% of all bacterial meningitis cases in the United States and is the primary cause of bacterial meningitis in children younger than 5 years old, except for neonates up to 2 months (Centres for Disease Control and Prevention, 2022; Prasad et al., 2025). Clinical symptoms of pneumococcal meningitis may include headache, vomiting, lethargy, irritability, fever, and seizures. Systemic complications, including septic shock, intravascular coagulation, and organ failure, can occur. In 50% of pneumococcal meningitis survivors, neurological complications, including hearing deficit, seizures, and motor deficits, occur (Centres for Disease Control and Prevention, 2022). Infection is usually preceded by nasopharyngeal colonization by pneumococci, avoidance of mucosal entrapment, and evasion of the immune system. Invasive disease may occur following bloodstream invasion, inducing the activation of complement and coagulation systems (Koedel et al., 2002). Pneumococci associated with the microvasculature can also cross the blood–brain barrier via a transcellular and paracellular pathway or migrate via the olfactory neurons into the brain, facilitated by inflammatory mediators (Koedel et al., 2002; Gil et al., 2023; Farmen et al., 2024). They can also invade the central nervous system from adjacent foci of infection. All brain regions are equally affected during disease progression, and the bacterial load in the brain is high with disruption of the blood–brain barrier enabling further invasion (Farmen et al., 2024). In the brain, the presence of bacteria leads to the activation of microglial cells, which can act as antigen-presenting cells, and recruitment of neutrophils, mediating an intense inflammatory reaction that is accompanied by the symptoms described above (Koedel et al., 2002). Pneumococcal virulence factors contribute to triggering the excessive inflammatory response, particularly the cholesterol-dependent cytotoxin pneumolysin, which is released upon bacterial lysis and also reduces motility of microglia (Hupp et al., 2019; Hupp et al., 2022). Because the bacteriolytic antibiotics used in the current standard of care of pneumococcal meningitis act by lysing the bacteria, they increase the amount of proinflammatory virulence factors such as pneumolysin released. To avoid the damage caused by this overwhelming inflammatory response, non-bacteriolytic antimicrobials have gained attention as potential new therapeutics to avoid lysing the bacteria and so avoid triggering the inflammatory response. These include the use of the non-bacteriolytic antibiotic daptomycin (Koedel et al., 2002; Cottagnoud et al., 2004; Grandgirard et al., 2007). Studies in animal models and in humans have found that the outcome of bacterial meningitis correlates with the severity of inflammation. Suppressing inflammation using agents such as complement inhibitors, matrix-metalloproteinase inhibitors, and non-bacteriolytic antibiotics such as daptomycin and the immunosuppressive corticosteroid dexamethasone has been investigated experimentally as a way of protecting the brain from excessive inflammation during pneumococcal meningitis (Tauber et al., 1985; Low et al., 1995; Mook-Kanamori et al., 2011; Bewersdorf et al., 2018). Despite recent advances, the mortality and morbidity rates for pneumococcal meningitis remain high and investigation of new non-bacteriolytic treatment options is important (Koelman et al., 2022; Müller et al., 2022; Ozisik, 2025).

In a recent study that characterized peptides released by *Klebsiella pneumoniae* in culture, an 11-amino acid peptide was identified, which was named V11A. This peptide was found to be taken up by *S. pneumoniae* specifically via its Ami permease, resulting in the inhibition of pneumococcal growth. V11A inhibited the growth of diverse *S. pneumoniae* strains, including those that were antibiotic resistant, in a bacteriostatic and species-specific manner in defined media (Lux et al., 2024). The V11A peptide also suppressed the growth of a trimethoprim-sulfamethoxazole-resistant clinical pneumococcal isolate added to human cerebrospinal fluid (hCSF). In addition, it reduced pneumococcal adherence to primary human airway epithelial cell cultures and reduced colonization of the rat nasopharynx, without causing any apparent toxicity to the eukaryotic cells (Lux et al., 2024).

Since peptide V11A has been shown previously to have a bacteriostatic effect *in vitro*, we hypothesized that it has potential as a novel therapeutic for pneumococcal meningitis. This is due to its action in curbing pneumococcal growth without causing bacterial lysis and therefore avoiding the release of virulence factors that trigger inflammation and worsen disease.

## 2 Results

### 2.1 Peptide V11A inhibits pneumococcal growth in hCSF in spiked samples and in hmCSF from a patient with meningitis

**Figure 1:**
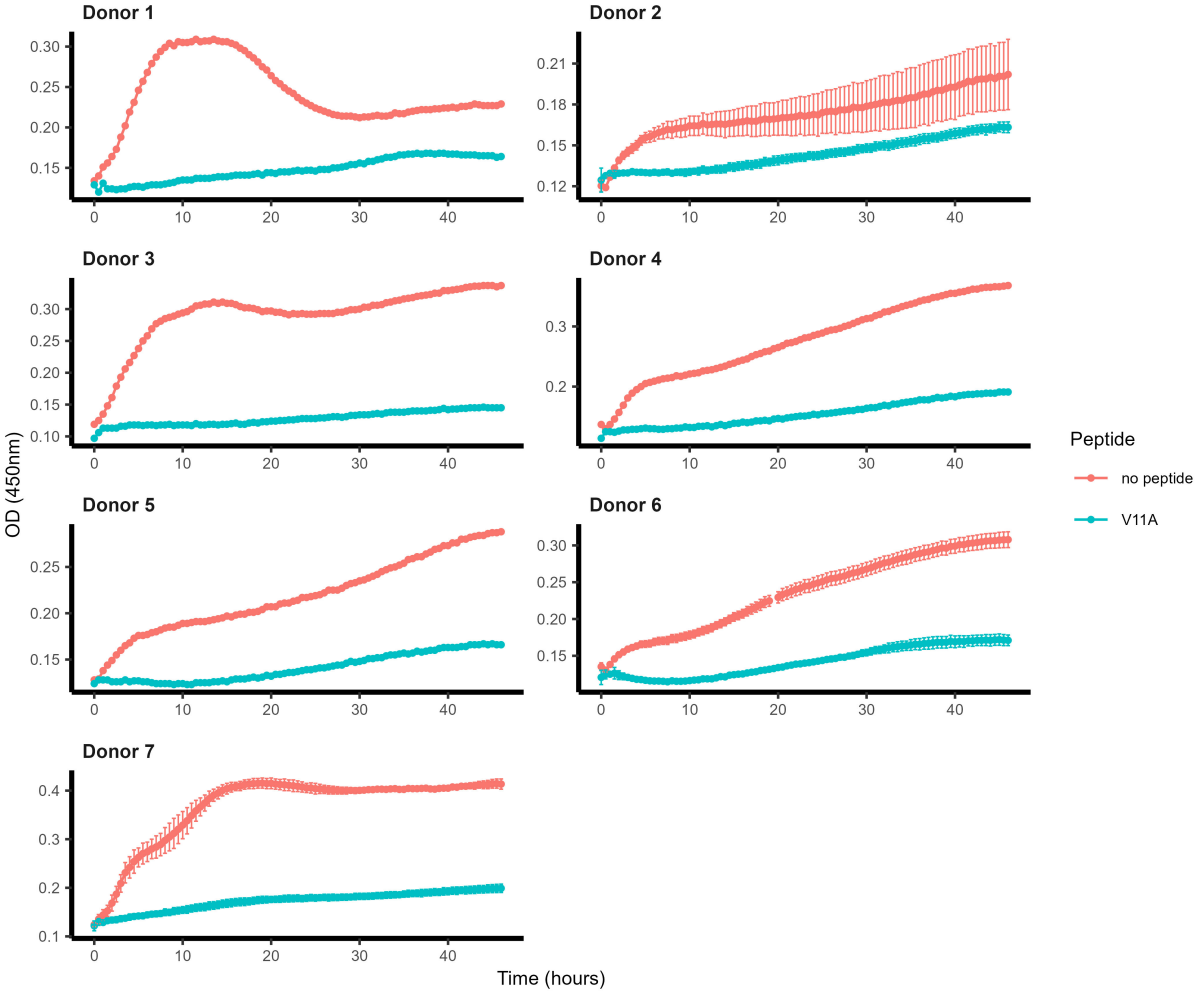
Effect of peptide V11A on growth of pneumococci spiked into human cerebrospinal fluid. Growth curves of pneumococcal strain 106.66 (serotype 6B) in the absence and presence of peptide V11A (0.5 mg/mL, endotoxin-free, TFA-free, acetate salt) in hCSF. Growth was monitored in undiluted hCSF by optical density (OD) (450 nm) measurement. Results represent one measurement due to the limited available hCSF for donors 1, 3, 4 and 5. Results represent two independent experiments with a total of at least three technical replicates for donor 2, three technical replicates for donor 6, and at least two technical replicates for donor 7.

We have previously demonstrated that V11A inhibited the growth of serotype 23F strain 115.75 inoculated into leftover hCSF from patients with non-inflammatory conditions (Lux et al., 2024). We tested whether this is also the case for serotype 6B strain 106.66 spiked into hCSF from several patients with (donors 1 and 7) and without (all other donors) inflammatory conditions. We found that V11A inhibited the growth of strain 106.66 in hCSF *in vitro* regardless of the inflammatory state (**Figure 1**).

**Figure 2:**
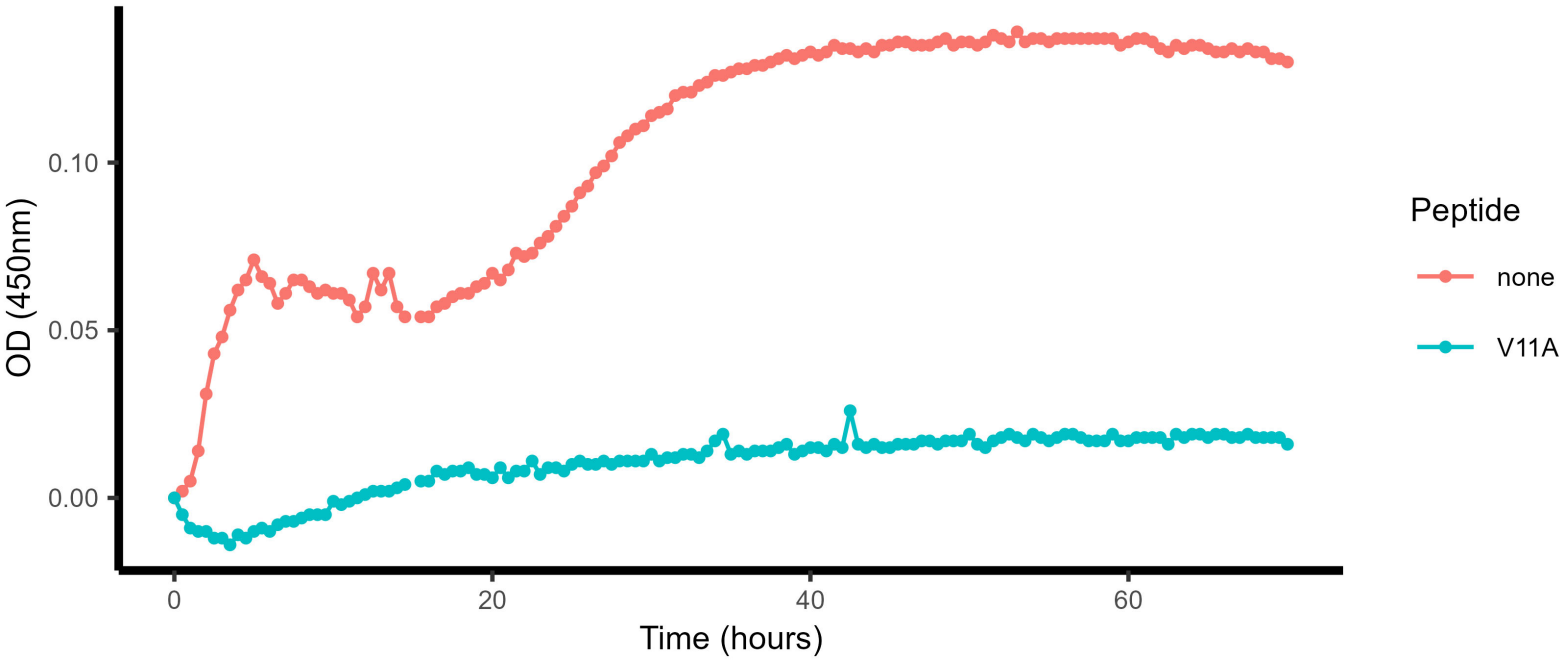
Effect of V11A on the growth of pneumococci present in the CSF of a patient with meningitis, *ex vivo*. Growth curve of pneumococci present in a fresh sample of human cerebrospinal fluid from a patient with pneumococcal meningitis (hmCSF) in the absence and presence of peptide V11A (0.5 mg/mL, TFA salt, not controlled for endotoxin). Background absorbance was corrected by subtracting the value of the first measurement. Results are from one patient due to the limited availability of fresh hmCSF.

Next, we tested the effect of V11A on the growth of pneumococci already present in hmCSF (*ex vivo*) from a patient with meningitis. *S. pneumoniae* was confirmed to be the only bacterial species present and was serotype 24F/B. We plated out dilutions of the sample onto Columbia sheep blood agar (CSBA) plates before performing the growth experiment and found that the sample contained 5.1 × 10^7^ cfu/mL pneumococci at the start of the growth experiment. We could observe that pneumococci initially present in the hmCSF sample grew in the absence of V11A, but that their growth was strongly inhibited by the presence of the peptide (**Figure 2**). In summary, V11A inhibited growth of pneumococci inoculated into hCSF, as well as already present in hmCSF.

### 2.2 Peptide V11A inhibits bacterial growth in pneumococcal meningitis *in vivo*

**Figure 3:**
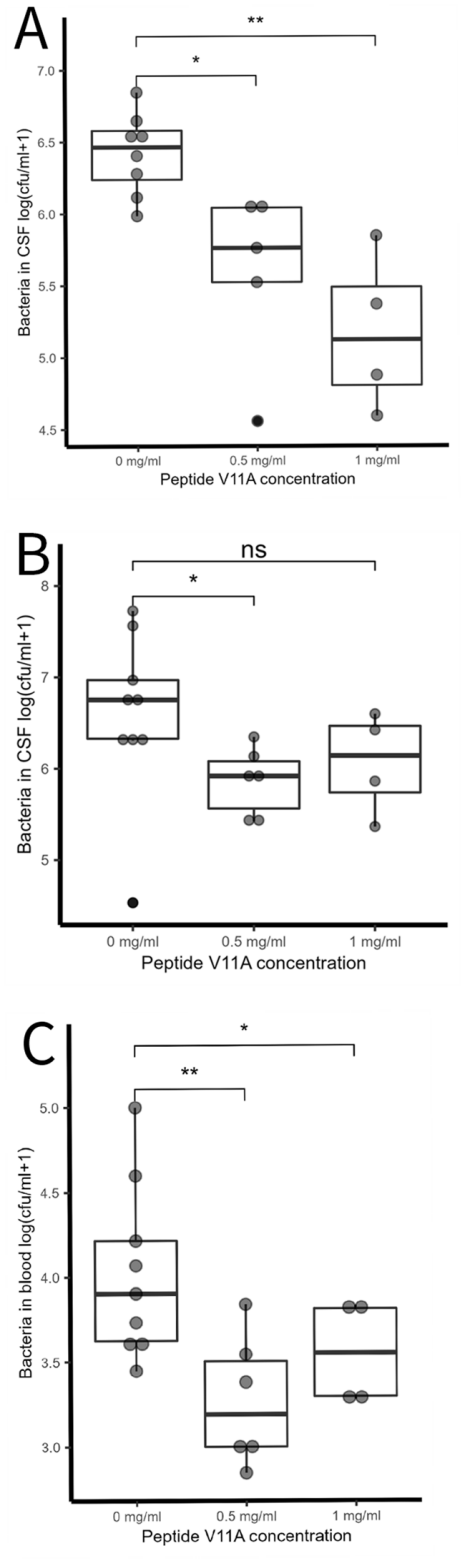
Effect of V11A on bacterial load in pneumococcal meningitis *in vivo*. **(A)** Bacteria in CSF at 6 h post-infection (hpi). * indicates *p*-value = 0.018, ** indicates *p*-value = 0.0073 by one-sided *t*-test. Results represent two independent experiments with 0.5 mg/mL V11A and one experiment with 1 mg/mL V11A, therefore three independent experiments without V11A. **(B)** Bacteria in CSF at 18 hpi. * indicates *p*-value = 0.03 by one-sided *t*-test. Results represent one independent experiment with 0.5 mg/mL V11A and one experiment with 1 mg/mL V11A, therefore two independent experiments without V11A. **(C)** Bacteria in blood at 18 hpi. ** indicates *p*-value = 0.0033, * indicates *p*-value = 0.036 by one-sided *t*-test. For each experiment, one litter of 14–15 (equal number of male and female) nursing Wistar rats was used. Three independent experiments were performed on different occasions. In the first experiment, we used 0.5 mg/mL V11A and had timepoints 6 and 18 hpi; in the second experiment, we used 0.5 mg/mL V11A and had timepoint 6 hpi; in the third experiment, we used 1 mg/mL V11A and had timepoints 6 and 18 hpi. Therefore, results represent one experiment with 0.5 mg/mL V11A and one experiment with 1 mg/mL V11A, therefore two independent experiments without V11A. Each point represents one animal.

Next, we tested whether peptide V11A affects the course of pneumococcal meningitis in an infant rat model. We injected pneumococcal strain 106.66 without and with peptide V11A at different concentrations into the cisterna magna of rat pups and sampled free-flowing CSF at different timepoints after infection and harvested blood and brains at the terminal timepoint. We found that at 6 h post-infection (hpi), the number of bacteria in the CSF (cfu/mL) was significantly reduced in the presence of 0.5 mg/mL V11A (*p* = 0.018) and 1 mg/mL V11A (*p* = 0.0073) (**Figure 3A**). At 18 hpi, the number of bacteria in the CSF was still reduced in the presence of V11A, but statistical significance was only reached with 0.5 mg/mL (*p* = 0.03) (**Figure 3B**). The bacterial load in the blood at 18 hpi was significantly reduced in the presence of 0.5 mg/mL V11A (*p* = 0.0033) and 1 mg/mL V11A (*p* = 0.036) (**Figure 3C**). We did not observe any adverse effect of the peptide on the animals.

### 2.3 Peptide V11A reduces inflammatory cytokine concentrations in CSF

**Figure 4:**
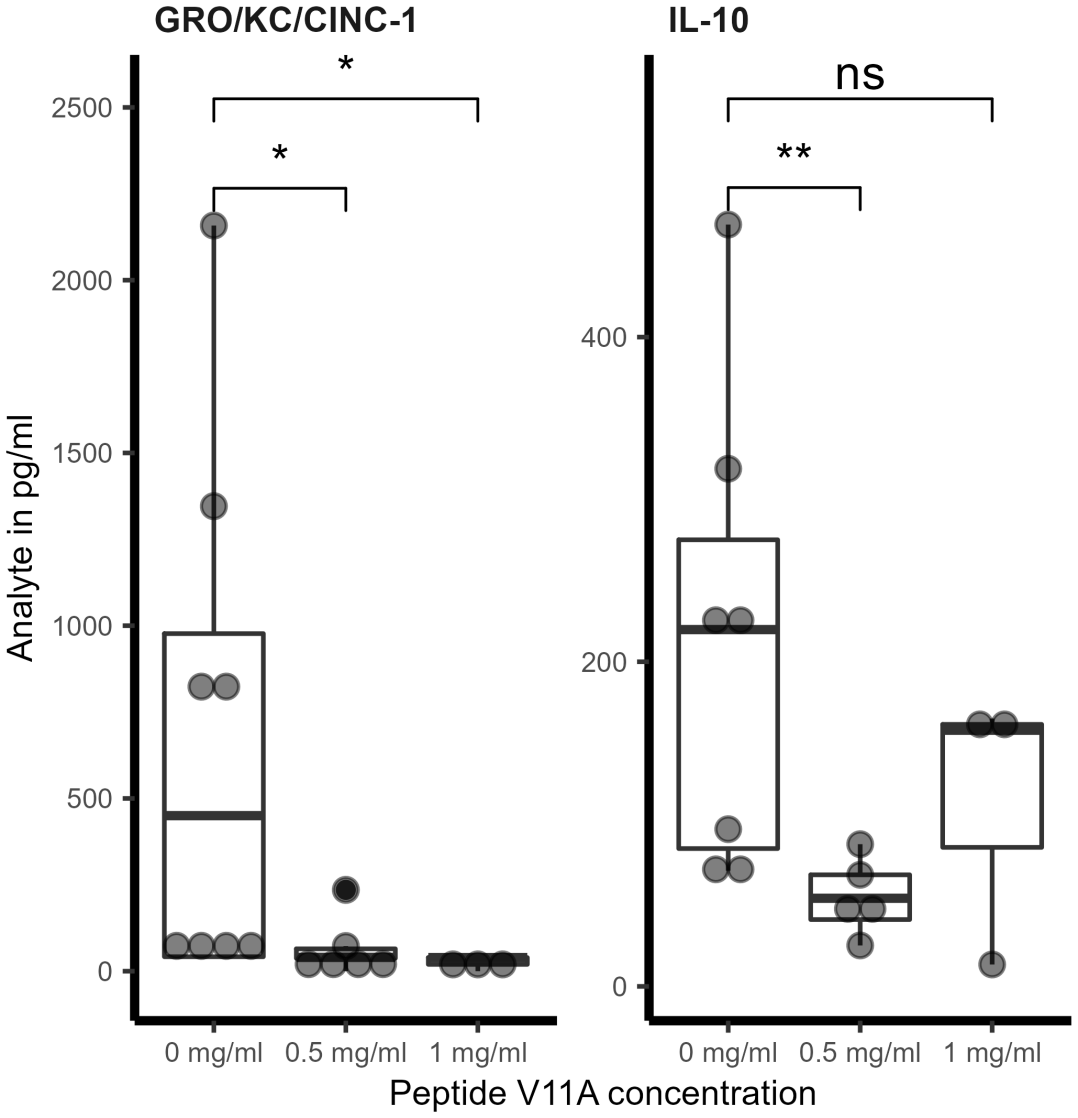
Effect of V11A on cytokine concentrations in CSF in pneumococcal meningitis *in vivo*. Cytokine concentration in CSF at 18 hpi. For GRO/KC/CINC-1, * indicates *p*-value = 0.04 for 0.5 mg/mL V11A and *p*-value = 0.024 for 1 mg/mL V11A compared to 0 mg/mL V11A calculated by one-sided Wilcoxon rank sum test. For IL-10, ** indicates *p*-value = 0.0051. Results represent one experiment with 0.5 mg/mL V11A and one experiment with 1 mg/mL V11A, therefore two independent experiments with 0 mg/mL V11A. Rat pups were infected with 10 μl of pneumococcal strain 106.66 (6B) intracisternally in 0.85% NaCl without or with peptide V11A (0.5 or 1 mg/mL). One point represents one animal. For cytokine measurements, out of range values at the lower detection limit were included in the analysis: the value corresponding to the detection limit provided by the manufacturer (IL-10 2.7 pg/mL and GRO KC CINC-1 19.7 pg/mL) was multiplied by the dilution factor used for the sample.

We measured cytokine concentrations in CSF samples taken at 6 and 18 hpi. As previously performed (Lux et al., 2024), samples under the detection limit were given a theoretical concentration corresponding to the lower detection limit provided by the manufacturer multiplied by the dilution factor for statistical purposes. At 6 hpi, we did not detect a significant effect of V11A on the concentration of any of the cytokines tested, although we did observe a trend of lower IL-6, IL-1 beta, and tumor necrosis factor (TNF) alpha concentration in the presence of V11A (**Supplementary Figure 1A**). At 18 hpi, V11A (0.5 and 1 mg/mL) significantly reduced the concentration of GRO/KC/CINC-1, an IL-8-like cytokine in rats (Lux et al., 2024). V11A (0.5 mg/mL at administration) also significantly reduced IL-10 concentration (**Figure 4**; **Supplementary Figure 1B**). We did not detect a significant effect of V11A on neurofilament light chain (NF-L), a biomarker for neuronal damage (Le et al., 2020; Chekrouni et al., 2022) (**Supplementary Figure 2A**). V11A did not significantly affect the number of apoptotic cells in the hippocampus, but we observed a trend to lower numbers in the presence of V11A (**Supplementary Figure 2B**).

### 2.4 Peptide V11A reduces inflammatory cytokine IL-8 concentration from human respiratory cells

**Figure 5:**
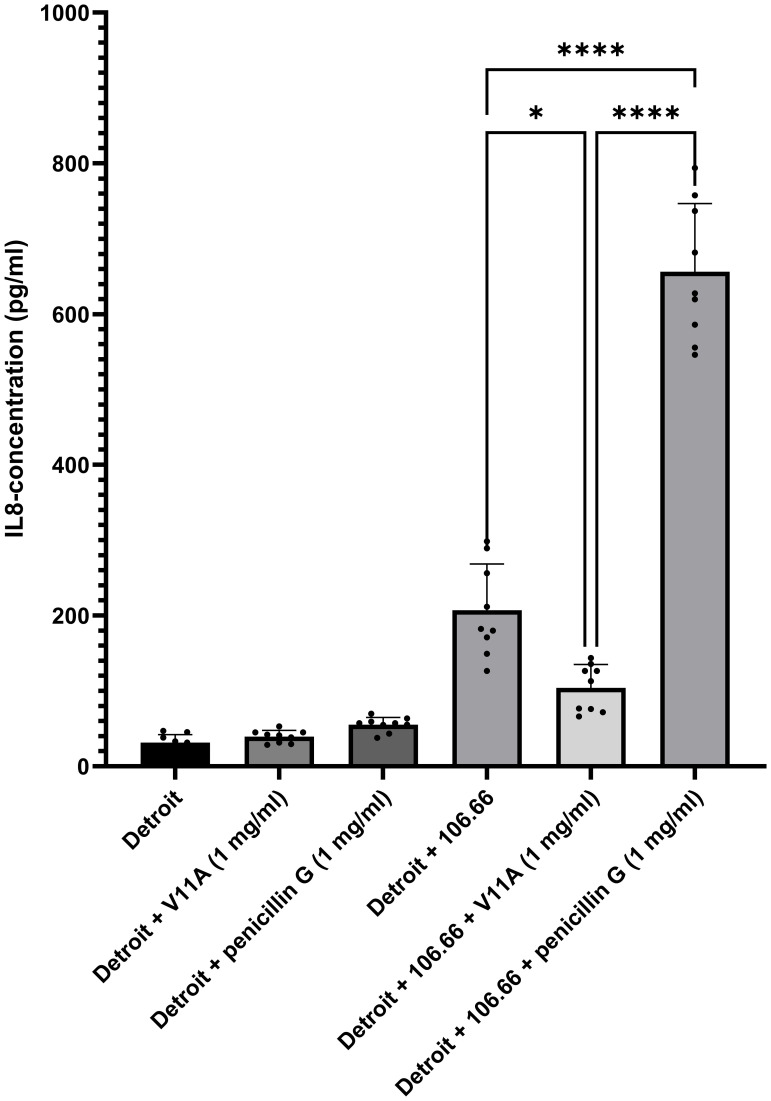
Effect of V11A in combination with *Streptococcus pneumoniae* 106.66 strain on IL-8 release from nasopharyngeal epithelial cells. Detroit 562 cells were incubated for 6 h in the presence or absence of *S. pneumoniae* strain 106.66 (serotype 6B) in the presence or absence of peptide V11A (1 mg/mL) or penicillin G (1 mg/mL, positive control). Detroit cells alone or with V11A only or with penicillin G only are negative controls. IL-8 levels in the culture supernatants were quantified by ELISA. Data represent the mean ± standard error of the mean (SEM) of three independent experiments, each performed in triplicate. Statistical significance was determined using one-way ANOVA with Šidák’s multiple comparison test. * indicates *p* = 0.011, **** indicates *p* < 0.0001.

To confirm the potential of V11A to reduce inflammatory cytokines released from host cells in response to *S. pneumoniae*, we assessed IL-8 secretion from the human nasopharyngeal epithelial cell line Detroit 562 (**Figure 5**). Pneumococcal strain 106.66 (serotype 6B) was added to Detroit cells in the presence or absence of either V11A (1 mg/mL) or penicillin G (1 mg/mL). The addition of 106.66 alone significantly increased IL-8 production, with an average concentration of 207.2 pg/mL, compared to the uninfected control (31.54 pg/mL). Notably, co-incubation of 106.66 with V11A significantly reduced IL-8 levels to 104.0 pg/mL (*p* = 0.0119), indicating that V11A attenuates pneumococcus-induced inflammation. Compared to V11A, the co-incubation of 106.66 with penicillin G significantly increased IL-8 levels to 656.1 pg/mL (*p* < 0.0001), indicating a potential advantage of V11A over lytic antibiotics in attenuating inflammation. However, it cannot be determined from this experiment whether the reduction in IL-8 release from the Detroit cells in the presence of V11A is due to an effect of V11A on host cell inflammatory signaling or whether it is due to modulation of bacterial activity, particularly growth. The effect was consistently observed across three independent experiments, each performed in triplicate. These findings support the potential of V11A to modulate host inflammatory signaling in response to pneumococcal challenge.

## 3 Discussion

Pneumococcal meningitis has a high mortality rate and a high rate of neurological sequelae in survivors due to the excessive inflammatory response, exacerbated by treatment with lytic antibiotics. We previously identified peptide V11A in the secretome of *K. pneumoniae* and discovered that it inhibits the growth of *S. pneumoniae* in a bacteriostatic and species-specific manner *in vitro* following specific uptake via the Ami permease (Lux et al., 2024). The peptide acts by downregulating genes involved in amino acid and protein metabolism, particularly branched-chain amino acid biosynthesis and transport as well as downregulating genes of the Ami permease itself. In addition, V11A reduced pneumococcal colonization *in vivo* (Lux et al., 2024). Here, the therapeutic potential of V11A in invasive pneumococcal diseases was tested using *in vitro* assays and a pneumococcal meningitis model to test its effect on bacterial load and inflammation.

Growth of pneumococci added to hCSF *in vitro* was inhibited by peptide V11A in samples from different donors regardless of whether or not the patients had inflammatory conditions, indicating that the peptide acts in more physiological environments as well as in culture medium.

Pneumococci thicken their capsule in rat CSF, and CSF from patients with meningitis contains cells, particularly neutrophils and lymphocytes, and has a high level of protein (Goonetilleke et al., 2010; Hathaway et al., 2016; Awulachew et al., 2020). To test whether V11A is still effective against pneumococci under conditions that more closely mimic those during disease, specifically the bacterial phenotype and the CSF composition, CSF was taken from a patient with pneumococcal meningitis (hmCSF). Pneumococci present in this fresh hmCSF grew in the absence of V11A, but growth was barely detectable in the presence of V11A, indicating that V11A was still effective under these conditions.

The potential of V11A as a therapeutic was also supported by the results *in vivo* in an infant rat meningitis model. V11A significantly reduced the number of bacteria in CSF 6 and 18 hpi and the number of bacteria in blood at 18 h. Therefore, the peptide still has an effect *in vivo* where CSF is constantly replenished at rate of at least 1 µL/min in rats (Takamata et al., 2001; Liu et al., 2020; Liu et al., 2022; Wichmann et al., 2022), which would diminish the local concentration of the peptide over time. In this proof-of-concept experiment, the peptide and bacteria were administered in the same injection; therefore, a limitation is that we cannot exclude that the peptide effect (partially) occurred in the inoculum.

V11A modulates the inflammatory response: it significantly reduced the concentration of cytokines GRO/KC/CINC-1 (an IL-8-like cytokine in rats (Himi et al., 1997)) and IL-10 in the CSF during experimental pneumococcal meningitis. It must be noted, however, that from our experiments, it cannot be determined whether V11A affects host cell inflammatory signaling or whether it is simply modulating bacterial activity. IL-8 is an important chemoattractant for neutrophils in humans (Spanaus et al., 1997) and has been found to be relevant in the pathogenesis of pneumococcal disease (Müller et al., 2021). IL-10 is an anti-inflammatory cytokine with diverse effects, including impairment of neutrophil phagocytosis and killing (Laichalk et al., 1996) and downregulation of proinflammatory cytokines and costimulatory molecules on macrophages (de Waal Malefyt et al., 1991; Thomssen et al., 1995). The ability of V11A to reduce the inflammatory response to *S. pneumoniae* was confirmed by measuring IL-8 release from a human nasopharyngeal cell line. While V11A reduced IL-8 release compared to cells treated with bacteria alone, treatment with the bacteriolytic antibiotic penicillin G greatly increased IL-8 release compared to cells exposed to bacteria alone or to bacteria with peptide V11A. We propose that the bacteriostatic action of the peptide, avoiding bacterial lysis and the subsequent release of proinflammatory factors, prevents an excessive inflammatory response, which could be a substantial advantage in the treatment of pneumococcal diseases, particularly meningitis, by reducing the inflammation, which leads to deficits in memory, learning, movement, and hearing. It is this dual effect of reducing not only bacterial numbers but also the damaging inflammatory response, and the fact that there are no directly comparable studies, which underlines the potential of V11A as a novel therapeutic against *S. pneumoniae.*

A limitation of the *ex vivo* study is that we only had fresh hCSF from one patient with pneumococcal meningitis due to the difficulty in obtaining samples. Another limitation of both the *ex vivo* and *in vivo* study is that we used high doses of peptide, although no adverse effects were observed *in vivo*. However, optimization by improving peptide delivery, activity, or stability may enable reduction of doses. As the therapeutic potential of V11A in pneumococcal meningitis was seen when administered together with the bacteria, this is a proof-of-concept experiment showing that V11A can be effective *in vivo* in pneumococcal meningitis. For application as a therapeutic, the peptide would need to be effective later during the development of the disease, when administered once infection and inflammation are established and symptoms appear, which may require peptide optimization.

In summary, synthetic peptide V11A, originally discovered in the secretome of *K. pneumoniae*, inhibited the growth of pneumococci in hCSF and reduced the number of bacteria and inflammation in an infant rat model of pneumococcal meningitis, when compared to untreated, infected animals. We propose that V11A has potential as a novel therapeutic agent against pneumococcal diseases due to its effectiveness at reducing the bacterial load via a bacteriostatic mechanism, thereby reducing the release of toxins, such as pneumolysin, and thus reducing the inflammatory response, which is very damaging to the patient with meningitis.

## 4 Methods

### 4.1 Peptides

V11A was initially identified in the secretome of *K. pneumoniae.* Here, the peptide used was synthetic and ordered with ≥95% purity (Genscript). For *in vivo* experiments, the peptide was endotoxin-free with TFA removed (salt form: acetate). Peptides were supplied as lyophilized powder and stored at −20°C and were used within 1 year. Peptides were resuspended in CDM or hCSF, according to the experiment, immediately before use. No peptide was returned to the freezer. The sequence of V11A peptide is VNATDEDRWNA.

### 4.2 Growth assays of pneumococci in human cerebrospinal fluid

Residual hCSF from seven anonymized patients undergoing lumbar puncture in 2023 was used for assays where pneumococci were inoculated into hCSF. Donors 2–6 underwent lumbar puncture due to non-inflammatory conditions as described previously (Lux et al., 2024) and donors 1 and 7 did the same due to inflammatory conditions. For the growth assay of bacteria present in hCSF, residual fresh hmCSF, positive only for the species *S. pneumoniae* (see below) from an anonymized patient with pneumococcal meningitis, was used. The identity of *S. pneumoniae* was confirmed from a culture by MALDI-TOF. Background absorbance was corrected by subtracting the value of the first measurement.

For the assays where hCSF was spiked with *S. pneumoniae*, strain 106.66 (serotype 6B) was stored at −80°C in the Protect Microorganism Preservation System (Technical Service Consultants Ltd.). After overnight culture on agar plates, 106.66 was sub-cultured in brain heart infusion (BHI) medium the next night until mid-log phase, corresponding to OD_600nm_ = 0.5. On the day of inoculation, 500 µL of the overnight culture was sub-cultured in 5 mL of BHI medium until OD_600nm_ = 0.5. Next, 5 mL was centrifuged at 3,000 *g* for 7 min, and the pellet was resuspended in 2.5 mL of phosphate-buffered saline (PBS, pH 7.4). The effect of peptide on the growth of 106.66 inoculated into hCSF from the different donors was measured based on the method of Brewster in sterile flat-bottomed 96-well microtiter plates (Thermo Fisher Scientific) (Brewster, 2003). The lid of the plate was treated with 2 mL of 0.05% Triton X-100 in 20% ethanol and dried before use to avoid condensation. In control wells, 200 µL of hCSF with 8 µL of bacterial inoculum was added, and in treatment wells, 200 µL of hCSF with 0.5 mg/mL peptide V11A and 8 µL of bacterial inoculum was added. The remaining wells were filled with 200 µL of PBS, and the plate was sealed with parafilm to avoid evaporation. OD_450nm_ was measured during incubation at 37°C every 30 min for 70 h in a plate reader (Nivo) with 5 s automatic shaking before each measurement. Growth curves were plotted in R.

The effect of peptide on the growth of pneumococci present in hmCSF from a patient with meningitis was measured as described above, without spiking the CSF. In one well, 200 µL of hmCSF was used as a control, and in another, 200 µL of hmCSF with 0.5 mg/mL peptide V11A was used. Background absorbance was corrected by subtracting the value of the first measurement. Growth curves were plotted in R. Serial dilutions of leftover hmCSF were plated onto CSBA plates and incubated overnight at 37°C and 5% CO_2_ before starting the growth assay, to determine initial bacterial concentration. The pneumococcal identity of the colonies grown on plates was also determined by MALDI-TOF (Bruker Microflex LT) and WGS sequencing (see the next section). The BIOFIRE FILMARRAY Meningitis/Encephalitis (ME) Panel (Biomérieux) was performed with the hmCSF sample prior to the experiment, confirming the detection of *S. pneumoniae* only. The panel also included *Escherichia coli K1*, *Haemophilus influenzae*, *Listeria monocytogenes*, *Neisseria meningitidis*, *Streptococcus agalactiae*, Cytomegalovirus, Enterovirus, Herpes simplex virus 1, Herpes simplex virus 2, Human herpesvirus 6, Human parechovirus, Varicella zoster virus, and *Cryptococcus neoformans/gattii*.

### 4.3 Bioinformatics

Genomic DNA was extracted from colonies of the *S. pneumoniae* strain isolated from the hmCSF with the QIAamp DNA Mini Kit (Qiagen) according to the manufacturer’s protocol. High-throughput sequencing (2 × 150 bp, 300 cycles) was performed using the Illumina DNA Prep Tagmentation assay using Nextera DNA CD Indexes on an Illumina MiSeq benchtop sequencer (Illumina) according to the manufacturer’s protocols at the Institute for Infectious Diseases, Bern. We used PneumoKITy v1.0 to determine the capsular type of *S. pneumoniae* using the paired-end raw sequencing reads and default parameters (Sheppard et al., 2022).

### 4.4 Pneumococcal meningitis infant rat model

Infecting organism: *S. pneumoniae* strain 106.66 (serotype 6B) was stored at −80°C in the Protect Microorganism Preservation System (Technical Service Consultants Ltd.). Strain 106.66 is a clinical isolate from a 4-year-old boy with otitis media in Switzerland (ST 2244, ID 3720). After overnight culture on agar plates, bacteria were sub-cultured in BHI medium overnight until mid-log phase, corresponding to OD_600nm_ = 0.5. On the day of infection, 500 µL of overnight culture was sub-cultured in 5 mL of BHI medium until OD_600nm_ = 0.4. After centrifugation at 3,000 *g* for 7 min and washing the bacterial pellet twice in 0.85% NaCl solution, the pellet was resuspended in 0.85% NaCl solution to obtain 5–7 × 10^6^ cfu/mL bacterial inoculum for the infection.

Animal model: We used an experimental model of pneumococcal meningitis in infant rats as previously described (Hathaway et al., 2016). For each experiment, one litter of 14–15 (equal number of male and female) nursing Wistar rats along with their dams (specific pathogen-free, SPF) were obtained from Charles River (Germany). Animals were acclimatized and kept housed in an individually ventilated cage (IVC) system under a 12-h light/dark cycle, maintained at a constant temperature of 22 ± 2°C, and given unrestricted access to tap water and gamma-sterilized pellet diet. Fourteen-day-old rat pups were infected by injection of 10 µL of 5–7 × 10^6^ cfu/mL bacterial inoculum with 0.5 or 1 mg/mL peptide V11A in sterile saline in the cisterna magna. Animals inoculated with bacteria without the peptide were used as control littermates. At non-terminal timepoints, free-flowing CSF was collected by puncture of the cisterna magna. At the terminal timepoint, all pups were sacrificed by an overdose of pentobarbital (Esconarkon, Streuli, Uznach, Switzerland; 150 mg/kg, i.p.), free-flowing CSF was taken when animals reached deep anesthesia, and blood was taken by cardiac puncture after thoracotomy. Animals were perfused with 4% paraformaldehyde in PBS and brains were collected. The CSF samples were directly processed by making a dilution series in PBS starting with 5 µL of CSF in the first dilution (1:100), and plating onto CSBA plates to determine cfu after an overnight incubation at 37°C in 5% CO_2_. The blood samples were directly processed by making a dilution series in 3.8% sodium citrate solution starting with 10 µL of blood in the first dilution (1:10), and plating onto CSBA plates to determine cfu after an overnight incubation at 37°C in 5% CO_2_. Residual samples that were not used for cfu determination were centrifuged at 16,000 *g* for 10 min at 4°C. Serum was prepared by letting the blood first coagulate for 30 min at room temperature before centrifugation at 16,000 *g* for 10 min. All supernatants were stored at −80°C until cytokine or NFL analysis. We performed three independent experiments on different occasions. In the first experiment, we used 0.5 mg/mL V11A and had timepoints 6 and 18 hpi; in the second experiment, we used 0.5 mg/mL V11A and had timepoint 6 hpi; in the third experiment, we used 1 mg/mL V11A and had timepoints 6 and 18 hpi.

### 4.5 Cytokine analysis

The levels of different cyto-/chemokines in rat CSF were measured with the magnetic multiplex bead-based assay MILLIPLEX^®^ Rat Cytokine/Chemokine Magnetic Bead Panel (RECYTMAG-65K, Millipore) and Bio-Plex200 System (Bio-Rad) for IL-6 and GRO KC CINC-1 (an IL-8-like cytokine in rats (Himi et al., 1997)) according to the manufacturer’s instructions. Up to 12.5 µL of CSF was used depending on sample availability from each rat pup. Values were normalized according to the sample dilution factor. The values corresponding to the detection limit provided by the manufacturer were as follows: IL-6, 30.7 pg/mL; TNF alpha, 1.9 pg/mL; IFN gamma, 6.2 pg/mL; IL-10, 2.7 pg/mL; IL-1 beta, 2.8 pg/mL; and GRO KC CINC-1, 19.7 pg/mL.

### 4.6 Neurofilament analysis of *in vivo* samples

The concentration of NFL was analyzed in the serum using an ELISA-based microfluidic system (ELLA, proteinsimple) and the SPCKB-PS-002448 V5 Human NFL kit (Bio-Techne) according to the manufacturer’s instructions. Serum was diluted 1:4 using 15 µL of serum for the analysis.

### 4.7 Determination of neuronal hippocampal apoptosis in rat brains

Neuronal apoptosis was quantified as previously described (Hathaway et al., 2016). Brains were cut at 45-µm intervals on a cryostat (Leica CM3050 S), coronal sections were mounted on gelatine-coated slides for Nissl staining with cresyl violet, and coverslips were fixed with DPX Mountant for histology (SIGMA Life Science). Cells with features of apoptosis were counted to assess neuronal apoptosis in the dentate gyrus of the hippocampus. Features included condensed, fragmented dark nuclei and apoptotic bodies. Apoptotic cells were counted in four slices spanning the hippocampus of both hemispheres, and a mean value was calculated per animal from counting cells in three fields of view in each of the two blades of the dentate gyrus in both hemispheres.

### 4.8 Detroit 562 cell culture

The human pharyngeal epithelial cell line Detroit 562 (ATCC CCL-138) was maintained under submerged conditions in complete Minimum Essential Medium (MEM) supplemented with 10% heat-inactivated fetal calf serum (FCS), 0.075% sodium bicarbonate, 1× MEM non-essential amino acids, 1 mM sodium pyruvate, 100 μg/mL streptomycin, and 100 U/mL penicillin (all from Gibco, Life Technologies, Switzerland). Cells were incubated at 37°C in a humidified atmosphere containing 5% CO_2_ and were passaged using 0.05% Trypsin-EDTA (Gibco, Switzerland) upon reaching 70%–90% confluence.

### 4.9 Analysis of IL-8 cytokine release *in vitro*

Detroit cells at 3 × 10^5^ in 1 mL of MEM without antibiotics were added to each well of a 24-well plate (TPP tissue culture plates, Sigma-Aldrich). The plate was incubated for 2 days at 37°C at 5% CO_2_. Monolayer integrity was confirmed by light microscopy. The culture medium was aspirated and replaced with 0.5 mL of assay medium consisting of a 1:1 mixture of MEM (lacking FCS and antibiotics) and a chemically defined RPMI-1640-based medium (Coleman et al., 2003) supplemented with 62 µM cysteine, 2.4 µM CuSO_4_, 148 µM MnSO_4_, and 5.78 mM glucose. *S. pneumoniae* strain 106.66 (serotype 6B) was grown overnight on CSBA plates and subsequently sub-cultured in 5 mL of BHI medium. Once mid-log phase (OD_600nm_ = 0.5) was reached, 500 µL of culture was transferred to fresh BHI (5 mL) and grown again to mid-log phase (OD_600_ = 0.5). Bacteria were harvested by centrifugation at 3,000 *g* for 7 min at room temperature. The supernatant was discarded, and the bacterial pellet was resuspended in 5 mL of assay medium. This washing step was repeated once more. The final bacterial suspension was diluted in assay medium to a concentration of approximately 3 × 10^6^ cfu/mL. Serial dilutions were plated on CSBA to verify the bacterial concentration retrospectively. Peptide V11A and penicillin G (control bacteriolytic antibiotic, Sigma-Aldrich) were each prepared at 1 mg/mL in assay medium. Medium from the Detroit cell cultures was aspirated, and 1 mL of assay medium (± V11A or penicillin G) or bacterial suspension (± V11A or penicillin G) was added to wells in triplicate. Plates were centrifuged at 120 g for 3 min at room temperature to enhance bacterial adherence and then incubated for 6 h at 37°C with 5% CO_2_. After incubation, supernatant was collected in 1.5-mL tubes and centrifuged at 16,000 *g* for 3 min at room temperature. Clarified supernatants were transferred to fresh tubes and stored at –80°C until further analysis. IL-8 concentrations were quantified using the Human IL-8/CXCL8 ELISA Kit – Quantikine (R&D Systems, Abingdon, United Kingdom), according to the manufacturer’s protocol. The minimum detectable dose of human IL-8 with this kit is 3.5 pg/mL. All assays were performed in triplicate, with three independent experimental replicates.

### 4.10 Statistics

Statistical analyses were performed in R version 4.0.3 within R studio version 1.3.1093. Shapiro–Wilk normality tests were performed to evaluate normality. If more than one sample group was not normally distributed, Wilcoxon rank sum test was applied; otherwise, *t*-test was used. For **Figure 5**, one-way analysis of variance (ANOVA) was performed. In all analyses, a *p*-value of ≤0.05 was considered statistically significant.

### 4.11 Ethics

All research adhered to applicable guidelines and regulations. In agreement with the Swiss Human Research Law, researchers at Bern University Hospital obtained anonymized discarded leftovers of hCSF samples for experiments in **Figure 1**, which had been collected in clinical routine. In compliance with the Swiss Human Research Law, researchers at Bern University Hospital obtained anonymized discarded leftover of hCSF collected in clinical routine from a patient with pneumococcal meningitis for the experiment in **Figure 2**. The discarded leftover sample was no longer needed for diagnostic purposes. Rat studies were approved by the Animal Care and Experimentation Committee of the Canton Bern, Switzerland (license number BE64/2022), and followed the ethical principles and guidelines for experiments on animals as published by the Swiss Federal Veterinary Office.

## Data Availability

The original contributions presented in the study are publicly available. The data can be found here: Sample accession: ERS28440733.
