# Supplementary material for: Synthetic peptide V11A reduces bacterial load and inflammation in pneumococcal meningitis
Source: Front Cell Infect Microbiol. 2026 Feb 9;15:1686525. doi: 10.3389/fcimb.2025.1686525 (PMC12926411; doi:10.3389/fcimb.2025.1686525)
Supplement: Supplementary file 1 [file DataSheet1.pdf]

SUPPLEMENTARY FIGURES

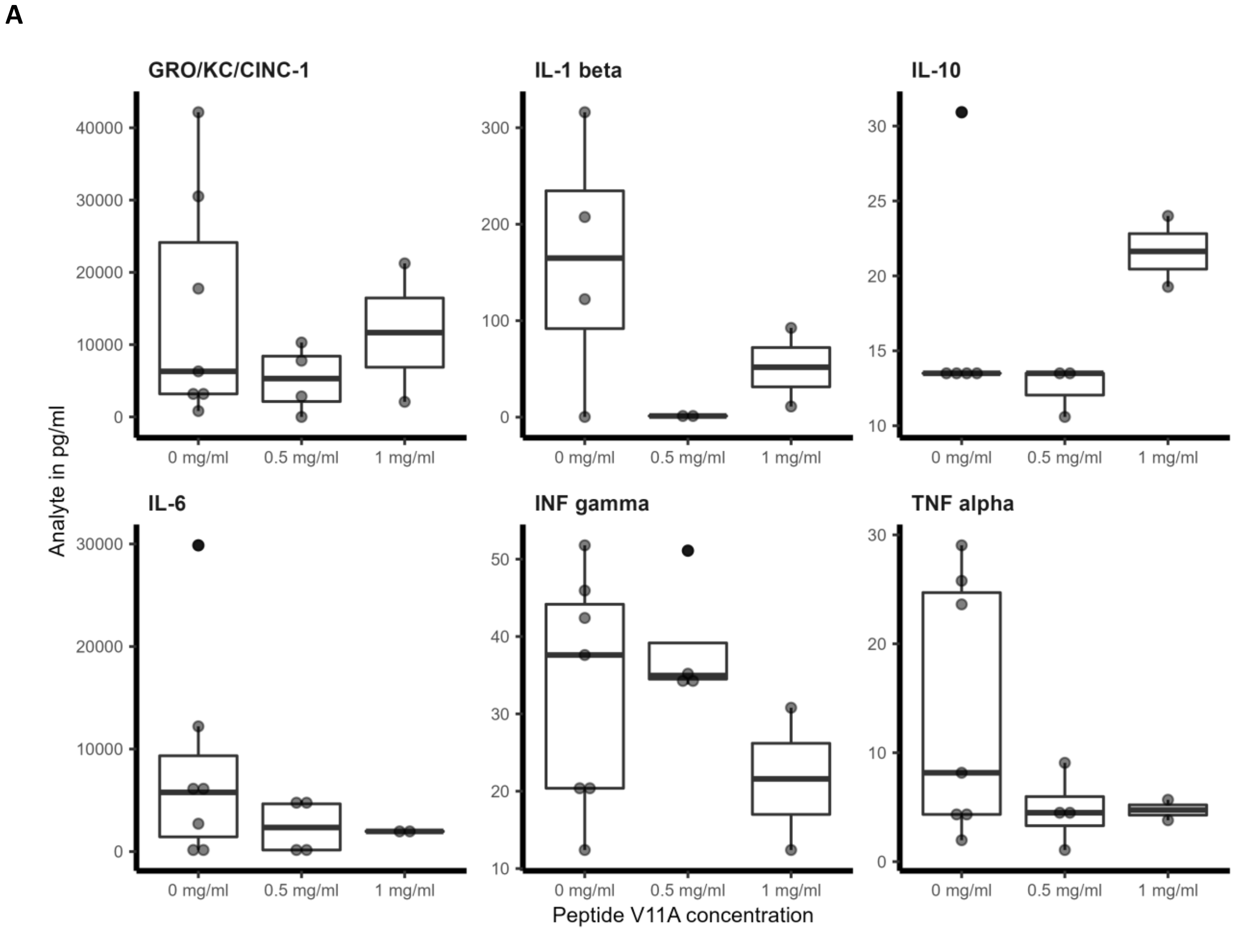

B

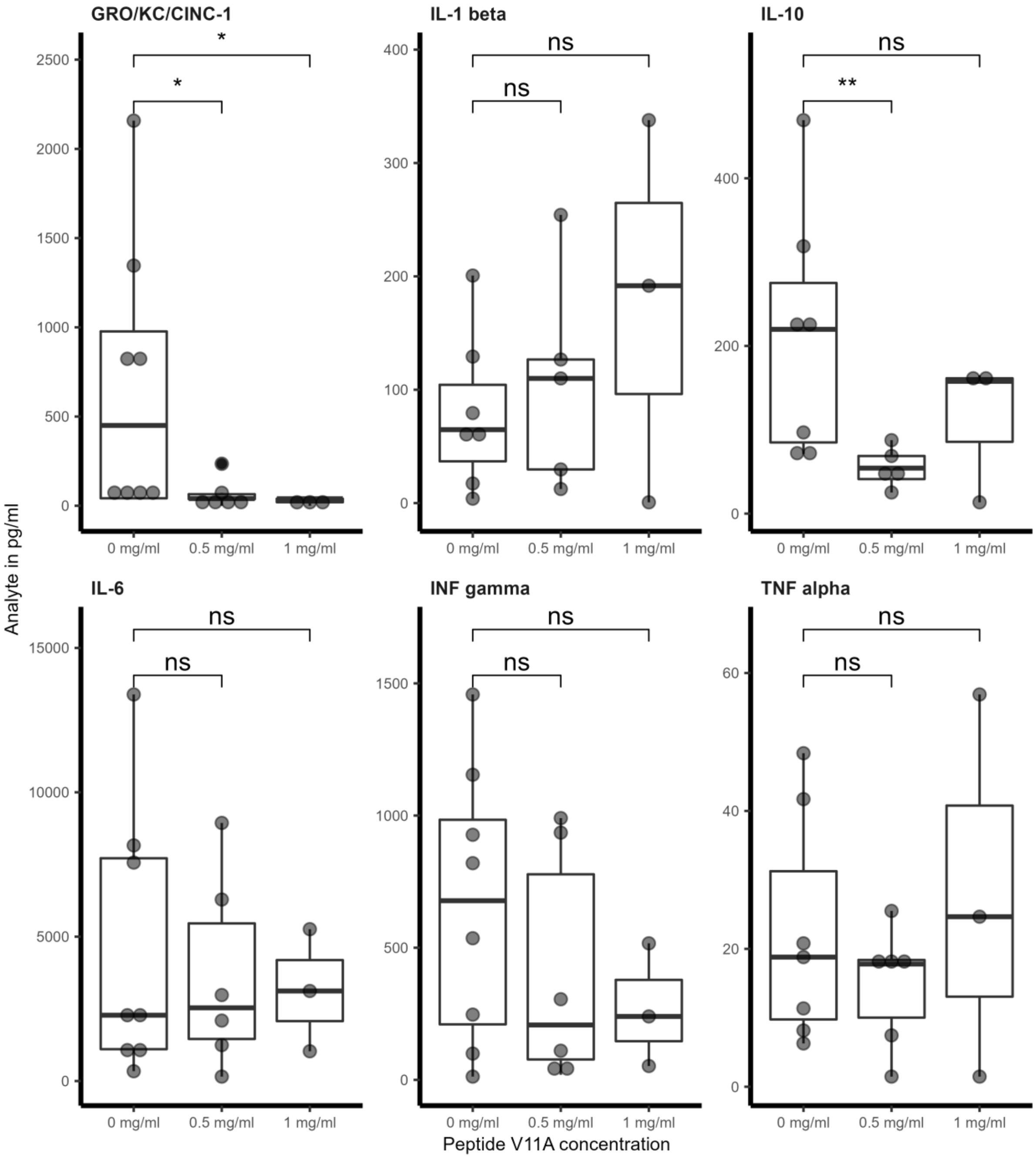

Supplementary Figure 1: Effect of V11A on cytokine concentrations in CSF in pneumococcal meningitis in vivo. A Cytokine concentration in CSF at 6 h post-infection (hpi). Results represent 2 independent experiments with 0.5 mg/ml V11A and 1 experiment with 1 mg/ml V11A, therefore 3 independent experiments with 0 mg/ml V11A. No significance detected by Wilcoxon rank sum test. **B** Cytokine concentration in CSF at 18 hpi. For GRO/KC/CINC-1 * indicates p value = 0.04 for 0.5 mg/ml V11A and p value = 0.024 for 1 mg/ml V11A compared to 0 mg/ml V11A calculated by one-sided Wilcoxon rank sum test. For IL-10 ** indicates p value = 0.0051. Results represent 1 independent experiment with 0.5 mg/ml V11A and 1 experiment with 1 mg/ml V11A, therefore 2 independent experiments with 0 mg/ml V11A.

Rat pups were infected with 10 µl pneumococcal strain 106.66 (6B) intracisternally in 0.85% NaCl without or with peptide V11A (0.5 mg/ml or 1 mg/ml). One point represents one animal. For cytokine measurements, out of range values at the lower detection limit were included in the analysis as done previously[1]: the value corresponding to the detection limit provided by the manufacturer (IL-6 30.7 pg/ml; TNF alpha 1.9 pg/ml; IFN gamma 6.2 pg/ml, IL-10 2.7 pg/ml, IL-1 beta 2.8 pg/ml; and GRO KC CINC-1 19.7 pg/ml) was multiplied by the dilution factor used for the sample.

Reference

1 Muller, A. *et al.* Meningitis-associated pneumococcal serotype 8, ST 53, strain is hypervirulent in a rat model and has non-haemolytic pneumolysin which can be attenuated by liposomes. *Front Cell Infect Microbiol* **12**, 1106063, doi:10.3389/fcimb.2022.1106063 (2022).

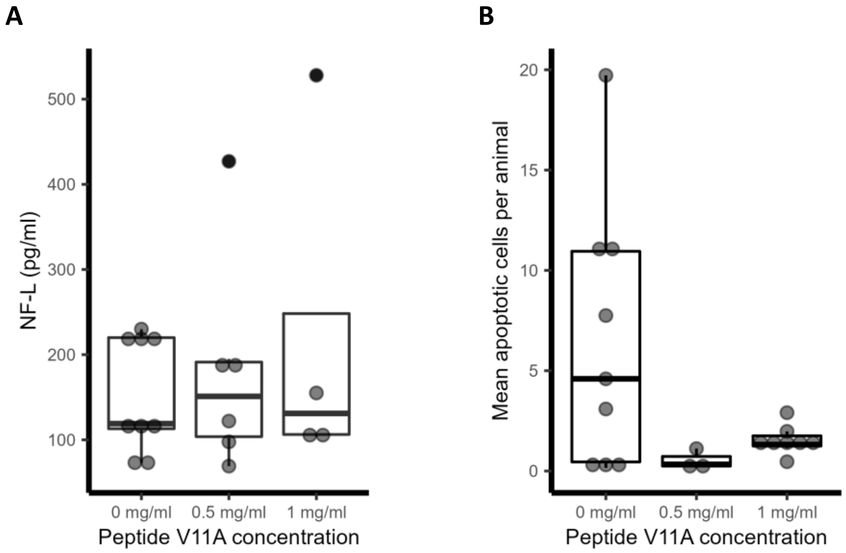

Supplementary Figure 2: Effect of V11A on markers of pneumococcal meningitis at 18 hpi in vivo. A Neurofilament light chain (NF-L) concentration in serum at 18 hpi. Two-sided Wilcoxon rank sum test showed no significance. **B** Number of apoptotic cells counted in the hippocampus. One-sided Wilcoxon rank sum test showed no significance.

One point represents one animal.
